# Effects of microgravity on osteoblast mitochondria: a proteomic and metabolomics profile

**DOI:** 10.1038/s41598-017-15612-1

**Published:** 2017-11-13

**Authors:** Anna Michaletti, Magda Gioia, Umberto Tarantino, Lello Zolla

**Affiliations:** 1 Department of Ecological and Biological Sciences, University of Tuscia, Viterbo, Italy; 2 Department of Clinical Medicine and Translational Science, University of Rome Tor Vergata, Rome, Italy

## Abstract

The response of human primary osteoblasts exposed to simulated microgravity has been investigated and analysis of metabolomic and proteomic profiles demonstrated a prominent dysregulation of mitochondrion homeostasis. Gravitational unloading treatment induced a decrease in mitochondrial proteins, mainly affecting efficiency of the respiratory chain. Metabolomic analysis revealed that microgravity influenced several metabolic pathways; stimulating glycolysis and the pentose phosphate pathways, while the Krebs cycle was interrupted at succinate-fumarate transformation. Interestingly, proteomic analysis revealed that Complex II of the mitochondrial respiratory chain, which catalyses the biotransformation of this step, was under-represented by 50%. Accordingly, down-regulation of quinones 9 and 10 was measured. Complex III resulted in up-regulation by 60%, while Complex IV was down-regulated by 14%, accompanied by a reduction in proton transport synthesis of ATP. Finally, microgravity treatment induced an oxidative stress response, indicated by significant decreases in oxidised glutathione and antioxidant enzymes. Decrease in malate dehydrogenase induced a reverse in the malate-aspartate shuttle, contributing to dysregulation of ATP synthesis. Beta-oxidation of fatty acids was inhibited, promoting triglyceride production along with a reduction in the glycerol shuttle. Taken together, our findings suggest that microgravity may suppress bone cell functions, impairing mitochondrial energy potential and the energy state of the cell.

## Introduction

Since space flight began, alterations in gravity (hypergravity and/or microgravity) represent a powerful physical cue for modelling both anatomy and function of living organisms^1,2^. Exposure to altered hyper-gravity can cause detrimental effects on muscle mass, composition, and contractility, as well as on bone density, with long-term effects even after return to normal gravity^3^. Moreover, hypergravity affects myoblast proliferation and differentiation^4^, PC12 neuron-like cell differentiation *in vitro* ^5^, cyclooxygenase-2 expression in the heart vessels *in vivo* ^6^, and the immune system^7^. On the other hand, the absence of gravity is also an extreme biological stressor and its impact on biological systems is ill-defined. Microgravity has the capability of inducing decreased immune function, bone density loss, and skeletal muscle atrophy and affects cardiac physiology of astronauts on off-planet missions^8,9^.

*In vivo*, the most severe effects on astronauts relate to reduction in bone mass and osteopenia, which accompany extended spaceflight^10^. Predominantly, microgravity induces pleiotropic effects in several tissues; bone, immune and nervous system, and skeletal muscle cells rearrange their cytoskeletal organisation and protein turnover, directing cells toward apoptotic death or premature senescence, altering cellular division and differentiation^11,12^. It is well documented that bone mineral density (BMD) of astronauts decreases at specific weight-bearing sites during spaceflight^13^, suggesting that osteoclasts and osteoblasts are gravitationally sensitive^14^. Gravitational biology studies report that gravitational force is a key factor in tissue construction^15^ and bone remodelling^16,17^. The response of biological cells to microgravity stressors is not only tissue-specific but also cell histotype-dependent. *In vitro*, osteoblastic cells are hypo-functional as, upon microgravity exposition, cells undergo alterations of cellular integrity with modified microtubule structure, focal adhesions and increasingly fragmented nuclei^18^. Conversely, osteoclast cells are hyper-stimulated by microgravity, displaying higher numbers of discrete resorption pits and increased cellular activity with an enhanced expression of mitochondrion-related genes and reduced roundness of mitochondria^14,19,20^. Mitochondria in skeletal muscle tissue can undergo rapid and unusual changes as a result of changes in muscle use and environmental conditions. Endurance exercise training can increase mitochondrial volume by up to 50% in previously untrained subjects^21^.

Although exhaustive physiological and cellular information on the response of osteoblast cells to microgravity has been collected, the gravitational stress-response proteins that are activated and mechanisms of the downstream effects are poorly understood. Thus, with the aim of revealing effects of microgravity on molecular processes beyond the cellular hypo-gravity response, two complementary mass spectrometry-based analytical ‘omics’ approaches were employed: proteomics and metabolomics. It is generally accepted that proteomics, based on mass spectrometry, allows for the relative quantitation of a large number of proteins concurrently and in a relatively unbiased manner^22^, while metabolomics information supports and corroborates the effects of the differential proteins found. Thus, a combination of two omics techniques, proteomics and metabolomics, was used to investigate the energy homeostasis of human cells exposed to simulated microgravity. Overall, this investigation drew an exhaustive picture of how mitochondria in human primary osteoblasts are functionally dysregulated by gravitational unloading.

We believe these findings will contribute to a more complete understanding of the cell physio-pathological processes which accompany astronauts’ diseases. In our opinion, evaluation of the biological effects of microgravity on energy homeostasis would be of help in space medicine for developing countermeasures to assure safe and effective aerospace missions.

## Results

The commercialized systems of desktop Random Positioning Machine (RPM) were employed as a ground-based model for simulating microgravity (or near weightlessness) for purified human primary osteoblast cells. Primary cultures of human osteoblasts were obtained from the cancellous bone of healthy donors with high-energy femoral fracture.

**Figure 1:**
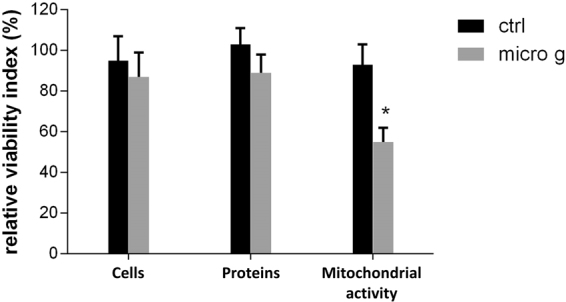
Effect of microgravity on viability of hpOB cells. The relative effect of microgravity treatment was expressed as relative cell viability index. The results were expressed as a percentage of: i) number of cells with intact plasma membrane (trypan blue impermeable cells), ii) cell protein content (BCA absorbance), and iii) metabolically active cells (MTS absorbance). Histograms represent mean ± SD (n = 6), black columns refer to control samples, whereas grey columns represent 110 hours-microgravity-exposed cells. Statistical significance was determined busing Student’s t-test. Significant decrease relative to respective control values at *p* < 0.05 is denoted as *.

First of all, we examined the macroscopic effects of simulated microgravity treatment on cellular biology, employing standard cell-biochemical assays. For each sample set, the relative cell viability was determined using three biochemical methods as follows: i) number of intact cells (*i*.*e*., number of trypan blue impermeable cells); ii) value of cell protein content (performed according to Biuret and BCA assay); and iii) formazan MTS formation (determined by colorimetric assay which detects the conversion of 3-(4,5-dimethylthiazol-2-yl)-2,5-diphenyltetrazolium bromide; MTT). With all three methods, the effect of gravitational unloading was calculated as the ratio of the mean value of treated group over the mean value of normogravity cells, expressed as a percentage. Figure 1 shows that 110 hours of microgravity treatment did not appear to alter human primary osteoblast (hpOB) proliferation, as the number of intact cells and cell protein content in the sample exposed to microgravity showed a slight decrease with respect to the normogravity cells. Regarding the mitochondrion-cell functionality, results of the formazan MTS assay showed that with microgravity there was a significant decrease in biocellular reduction efficiency (*p* < 0.05) (Fig. 1), thereby indicating that the prevalent impact of microgravity treatment affected mitochondrial metabolism rather than cell vitality. This apparent discrepancy was further investigated, focusing on mitochondrial metabolism using two “omics” approaches. Therefore, the hpOBs cells were exposed to simulated microgravity for 110 hours and then examined for proteomic and metabolomic changes.

### Proteomic profiling

**Figure 2:**
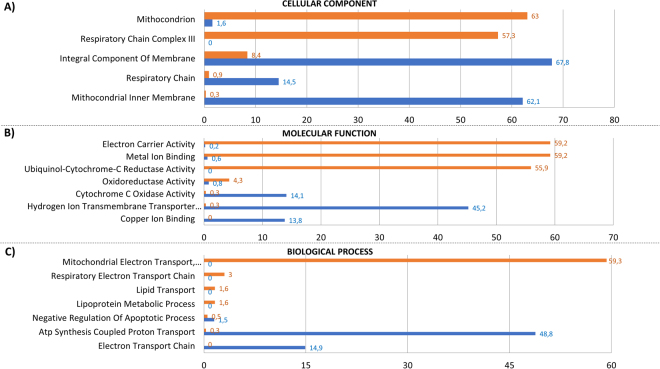
Functional enrichment analysis of hpOB under Normogravity and Microgravity using FunRich. Bioinformatics Gene Ontology-based classification of proteomes according to three categories: cellular component (**A**), molecular function (**B**), and biological process (**C**). Numbers next to the bars represent levels of normogravity (blue bars) and microgravity (orange bars), respectively. Only categories with >2-fold change are shown.

Purified protein extracts were eluted and separted through a 16–8% linear gradient polyacrylamide gel 1D SDS-PAGE and analyzed by nano-LC-MS/MS system (Supplementary Fig, S1). Proteome changes associated with microgravity were analysed using FunRich (Platform 3.0). As shown in the Venn diagrams, the identification of peptide sequences from the hpOB proteome generated 813 and 978 proteins from normogravity- and microgravity-exposed samples, respectively (562 proteins were in common between the two conditions) (Supplementary Fig. S2). The whole identified proteome was analysed first using Gene Ontology (GO) biological process annotations through FunRich and then using a stand-alone software tool which allows functional enrichment analysis of complex protein and gene datasets (Fig. 2). According to FunRich Bioinformatics analysis, the whole set of proteins could be classified according to categories based on three annotated biological processes: the cellular component (Fig. 2A), molecular function (Fig. 2B) and biological process (Fig. 2C). Figure 2 displays only those categories showing greater than a two-fold change, while the full set is shown in Supplementary Fig. S3.

Among all cellular components, Fig. 2 clearly shows that the mitochondrion was affected the most by microgravity. Over 50% of the hypogravity-sensible proteins are related to mitochondrial processes, including mitochondrial protein homeostasis (mitochondrial protein import) and energy production. In this paper, we focused our attention on those proteins involved in the respiratory chain, glycolytic metabolism and antioxidant enzymes; the remaining protein categories have been investigated in a subsequent study (paper in preparation).

With regard to the mitochondrial proteome, microgravity reduced the integral component of the membrane and, in particular, proteins of the inner membrane, such as the respiratory chain Complex IV (by about 14%), while Complex III increased by 60%. Table 1 shows that several mitochondrial enzymes were altered, suggesting that glycolysis, the PPP (pentose phosphate pathways) and Kreb’s cycle are sensitive to gravity. Among Kreb’s cycle enzymes, of particular note are succinate dehydrogenase (Complex II), fumarase (FUMH) and malate dehydrogenase (MHDM), were reduced by microgravity (50%; 53% and 62% respectively). Moreover, Table 1 listed the specific proteins belonging to Complex III and IV which were up or down-regulated. Finally, an additional interesting group concerned with antioxidant enzymes were listed which are involved in mitochondrion perturbation. This group includes glutathione peroxidase (GPX7), peroxiredoxin 4 (PRDX4), 5 (PRDX5) and 6 (PRDX6), superoxide dismutase (SODC) and glutathione peroxidase 8 (GPX8), all of which were over-expressed in the gravitational unloading environment. Interestingly, ROS production increased significantly (data not shown).

**Table 1:** Spectral count-based quantitation of proteins identified between the human osteoblasts under normogtavity and microgravity.

Molecular function enrichment item (FunRich)	Uniprot Protein ID	Protein Name	EmPAI quantification		p-Value	Trend
			mean_Normogravity	mean_Microgravity		
Glycolysis	TPI1	Triosephosphate isomerase	1,223948	0,641568	0,010695	DOWN (*)
	LDHA	L-lactate dehydrogenase A chain	0,399377	0,633762	0,000753	UP (***)
	G3P	Glyceraldehyde-3-phosphate dehydrogenase	0,9990652	0,0987282	0,000849	DOWN (***)
	ENOA	Enolase	1,15353047	0,70729455	0,020571	DOWN (*)
	PGAM1	Phosphoglycerate mutase 1	0,274401	0,329345	0,000607	UP (***)
Kreb’s cycle	ODO2	2-oxoglutarate dehydrogenase, mitochondrial	0,01746131	0,00829692	0,047496	DOWN (*)
	SUCB1	Succinyl CoA Synthetase	0,015458	0,009332	0,003271	DOWN (**)
	FUMH	Fumarase	0,017809	0,008353	0,035839	DOWN (*)
	SDHA	Succinate dehydrogenase	0.01593	0.007965	3,836E-05	DOWN (***)
	MDHM	Malate dehydrogenase	0,156028	0,059646	0,000105	DOWN (***)
PPP	6PGD	6-phosphogluconate dehydrogenase	0,015458	0,036229	0,002285	UP (**)
	TKT	Transketolase	0,174453001	0,17323428	0,070788	DOWN
Ubiquinol-cytochrome-c reductase activity	UQCRC1	Cytochrome b-c1 complex subunit 1, mitochondrial	0,015736	0,02952	5,39E-05	UP (**)
	NQO1	NAD(P)H dehydrogenase [quinone] 1	0,051803	0,122297	5,39E-05	UP (***)
	UCRI	Cytochrome b-c1 complex subunit Rieske, mitochondrial	0,028819	0,016934	0,007927	DOWN (*)
	CYB5R3	NADH-cytochrome b5 reductase 3	0,099498	0,154874	0,001792	UP (*)
Cytochrome c oxidase activity	COX5A	Cytochrome c oxidase subunit 5A, mitochondrial	0,117605	0,065708	0,008712	DOWN (*)
	CYB5A	Cytochrome b5	0,051882	0,034082	0,000974	DOWN (***)
	COX6C	Cytochrome c oxidase subunit 6C	0,088148	0,060621	0,032165	DOWN (*)
Antioxydant activity	PRDX1	Peroxiredoxin-1	0,792314	0,718062	0,296566	DOWN
	PRDX2	Peroxiredoxin-2	0,387978	0,36369	0,085143	DOWN
	PRDX3	Thioredoxin-dependent peroxide reductase, mitochondrial	0,12877	0,1721	0,000297	DOWN (***)
	PRDX4	Peroxiredoxin-4	0,140379798	0,149817616	0,683571	UP
	PRDX5	Peroxiredoxin-5 mitochandrial	0,133505475	0,237667115	0,000334	UP (***)
	PRDX6	Peroxiredoxin-6	0,326313	0,417195	0.000055	UP (***)
	SODC	Superoxide dismutase	0,122147741	0,164447199	0,014818	UP (*)
	GPX7	Glutathione peroxidase 8	—	0,024664838	—	UM
	GPX8	Glutathione peroxidase 7	—	0,017486587	—	UM

Protein content was estimated as percentage of normalized emPAI values, as reported in Shinoda *et al*. The table shows differentially regulated proteins in abundance with statistical significance. *p < 0.05; **p < 0.01; ***p < 0.001, and unique *p*roteins in normogravity (UN) and microgravity (UM).

### Metabolic profiling of osteoblasts under microgravity

To explore the microgravity-induced perturbations on hpOB cells, a quantitative metabolic comparison was carried out between metabolomes extracted from normogravity- and microgravity-cultured cells. Multivariate statistical analyses were employed for the HPLC-MS data sets. Using this approach, 137 metabolites detected across both groups met acceptability criteria and were further analysed using bioinformatics tools (MetaboAnalyst 3.0 software) (Supplementary Fig. S4).

Glycolysis, Kreb’s cycle, PPP, the glycerol-phosphate shuttle, as well the malate-aspartate shuttle, were all significantly reduced by microgravity (in agreement with proteomic analysis). Microgravity also influenced aspartate metabolism and galactose metabolism, which are apparently connected to mitochondrial dysfunction (see Discussion).

**Figure 3:**
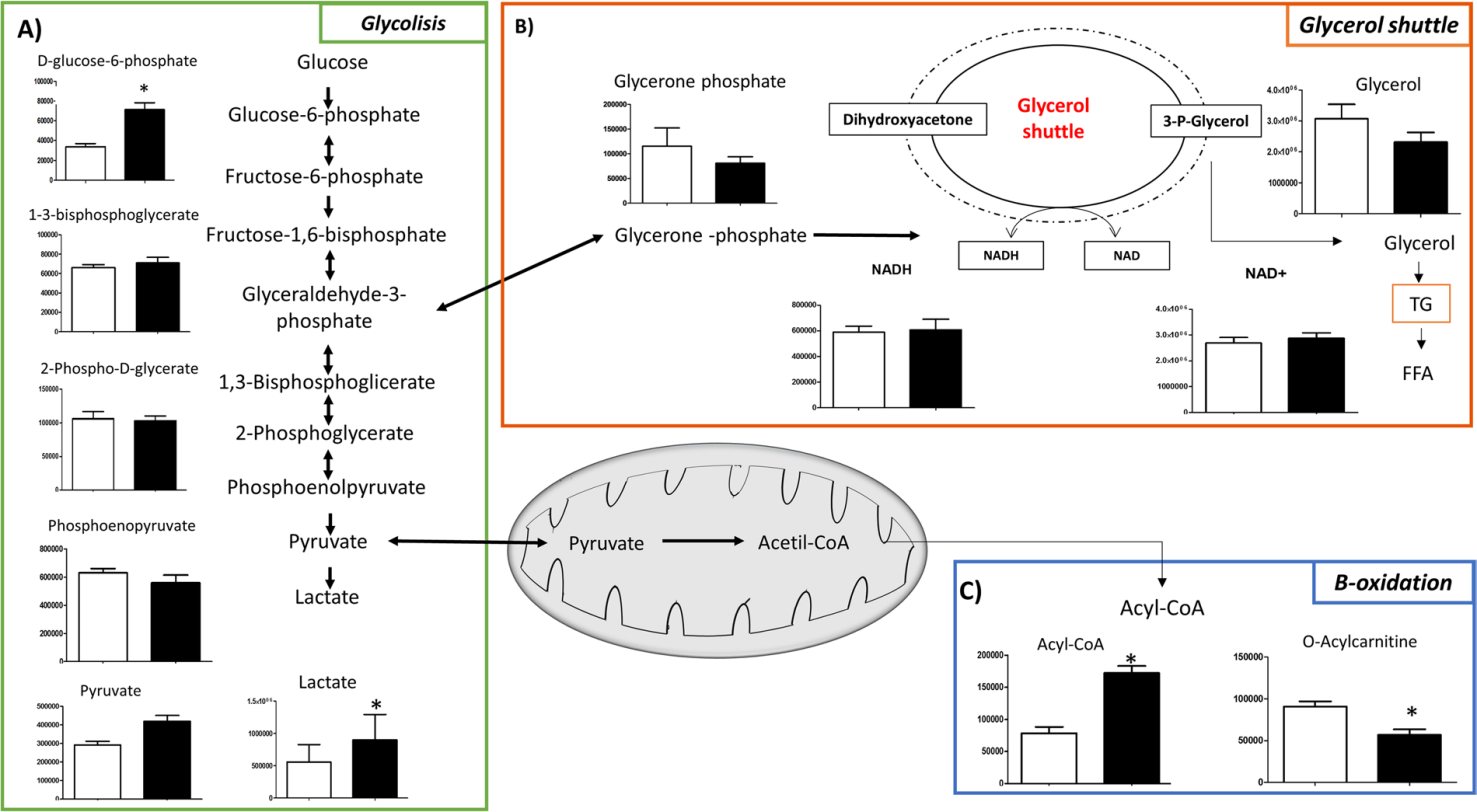
Metabolic cross-talk among glycolysis, glycerol shuttle and β-oxidation pathways. Variation in the levels of metabolic intermediates of: (**A**) glycolysis, (**B**) glycerol shuttle and (**C**) two intermediates of β-oxidation. Values represent mean ± SD (n = 9) of normogravity (white columns) and microgravity (black columns) metabolites. Statistical significance was indicated by *p < 0.05; **p < 0.01; ***p < 0.001.

Under conditions of microgravity, the glycolysis pathway was moderately stimulated, since glucose-6-phosphate increased as well as other glycolytic precursors (Fig. 3A). Interestingly, under microgravity conditions the amount of glycerone phosphate was reduced (Fig. 3B), suggesting that the triose equilibrium was displaced in favour of glyceraldehyde-3-phosphate, thereby reducing the glycerol shuttle (Fig. 3B). Further, under microgravity there was a decrease in acyl-carnitine, mirrored by an increase in acyl-CoA (Fig. 3C), suggesting decreased free fatty acid transport into mitochondria and a reduction in the beta oxidation pathway, in agreement with a recent study^23^.

**Figure 4:**
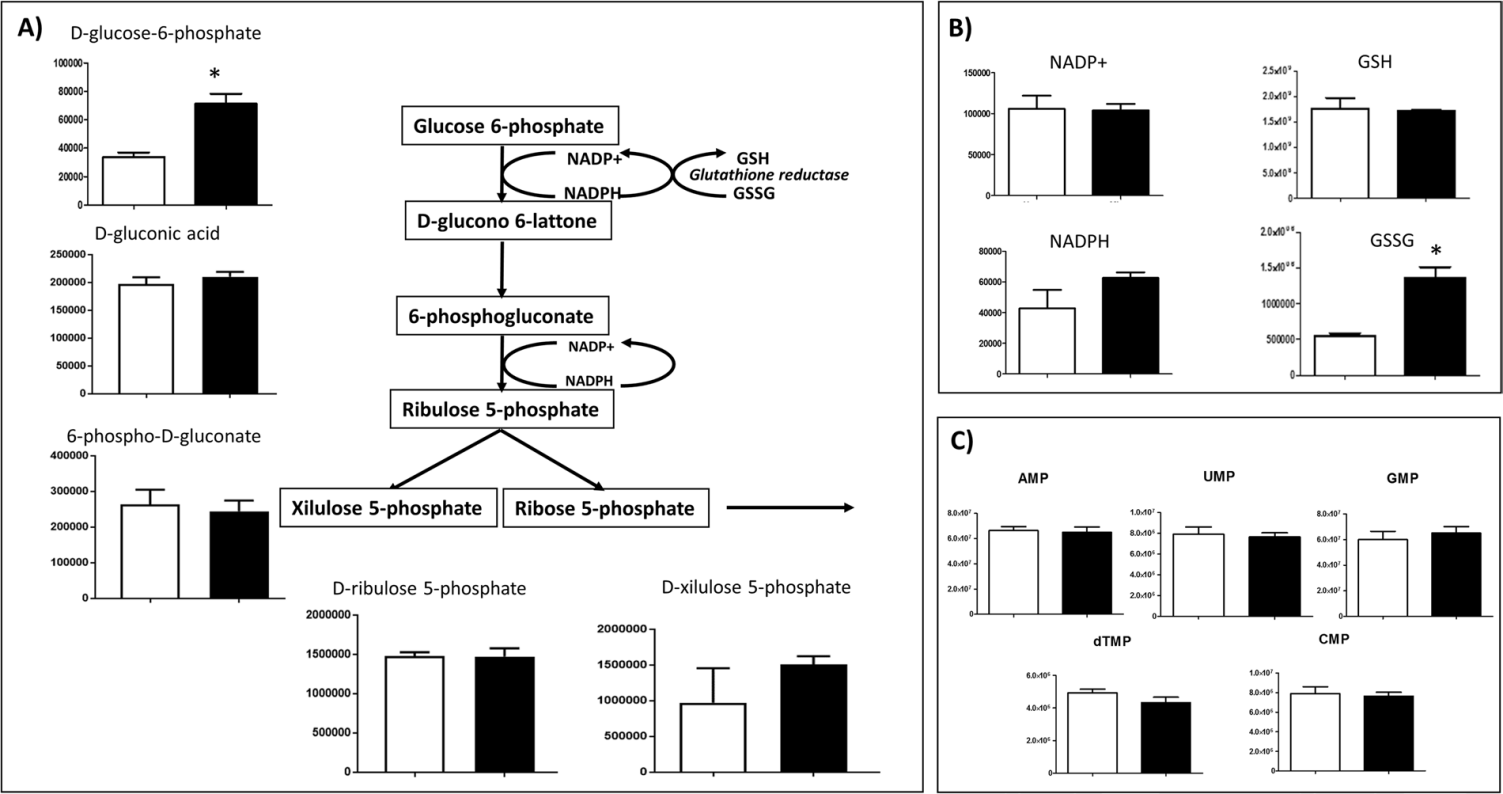
Intermediates of PPP pathway. Variation in the levels of metabolic intermediates in PPP cycle. Panel (A) represents NADP/ NADPH and GSH/GSSG. Panel (B) represents nucleotide monophosphate. Values are mean ± SD (n = 9) of normogravity (white columns) and microgravity (black columns) metabolites. Statistical significance was indicated by *p < 0.05; **p < 0.01; ***p < 0.001.

PPP turned out to be particularly stimulated under microgravity, as demonstrated by both the proteomic analysis (up-regulation of glucose-6-phosphogluconate lactone dehydrogenase, connected to the first step reaction; shown in Table 1) and the metabolomic analysis, where the product of the first reaction step (D-glucono-6-lactone) was more abundant (Fig. 4A). Concurrently, this produced increased levels of oxidised glutathione (GSSG) intermediate (Fig. 4B). These data, together with the up-regulation of glutathione peroxidases (Table 1), suggest that oxidative stress was induced by microgravity exposition. Interestingly, no accumulation of ribose-5-phosphate was recorded and, consequently nucleotide synthesis remained at a constant level (Fig. 4C). Thus, in contrast to cancer and dedifferentiated cells^24^, up-regulation of the glycolysis pathway under microgravity conditions did not stimulate cell replication.

**Figure 5:**
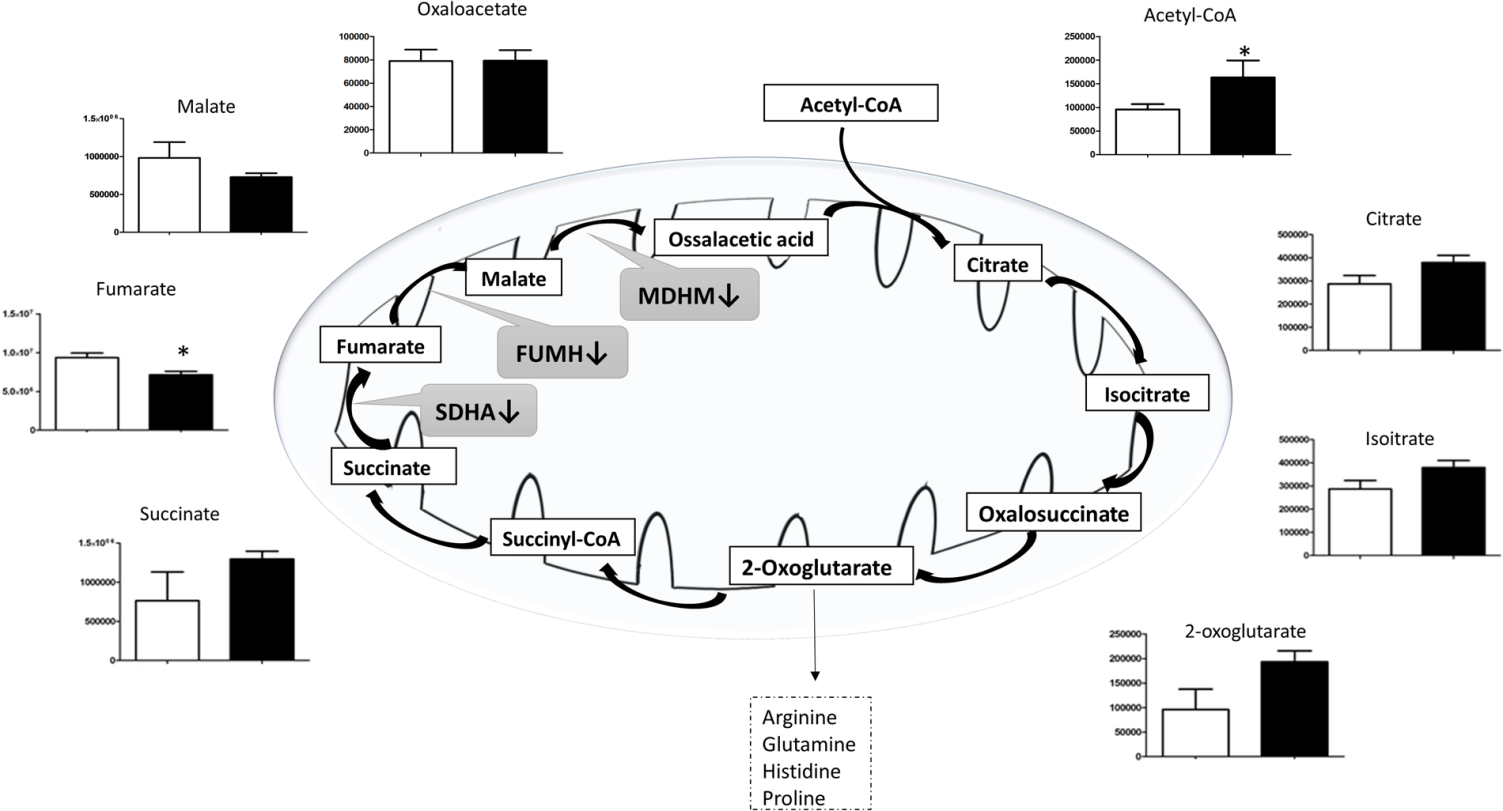
Intermediates of tricarboxylic acid pathway (TCA). Variation in the levels of metabolic intermediates in Kreb’s cycle. It was interrupted at succinate production with a decrease in fumarate and malate levels, and down-regulation of FUMH, MDHM and succinate dehydrogenase enzymes (black arrow). Values are mean ± SD (n = 9) of normogravity (white columns) and microgravity (black columns) metabolites. Statistical significance was indicated by *p < 0.05; **p < 0.01; ***p < 0.001.

**Figure 6:**
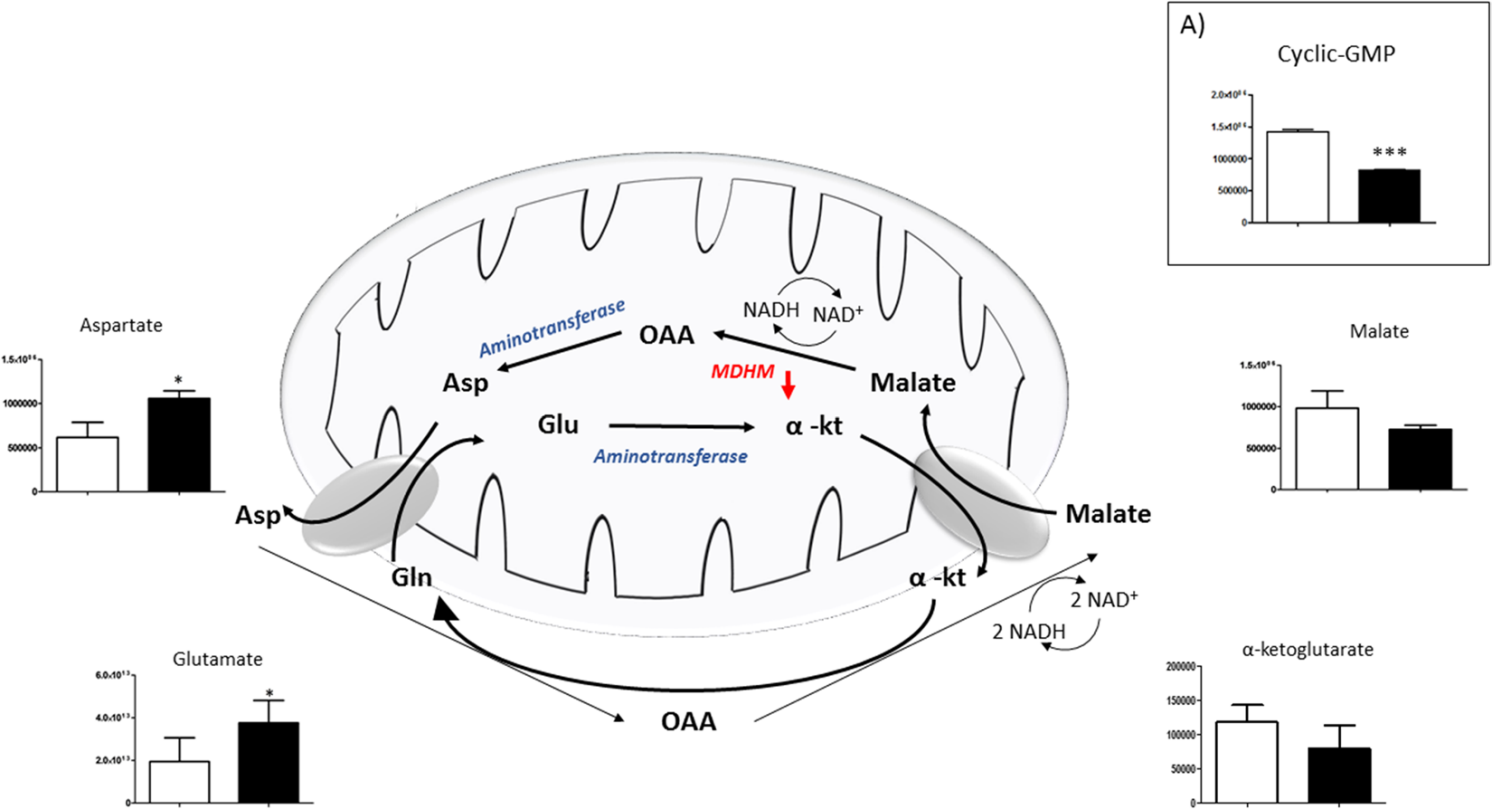
Intermediates of Malate-Aspartate Shuttle. Variation in the levels of metabolic intermediates of the malate-aspartate shuttle. Down-regulation of MDHM enzyme is represented by red arrow. Panel 6 (A) show the decrease of Cyclic-GMP. Values are mean ± SD (n = 9) of normogravity (white columns) and microgravity (black columns) metabolites. Statistical significance was indicated by *p < 0.05; **p < 0.01; ***p < 0.001. (OAA = Oxalacetate; Asp = Aspartate; Glu = Glutamate; α-kt = α-ketoglutarate).

Under microgravity, the Kreb’s cycle was activated, as indicated by acetyl-CoA accumulation. However, this stimulation only affected the first part of the cycle, showing an increase in products of the Krebs cycle, until succinate transformation into fumarate where the trend was reversed, with a consequent decrease in fumarate and malate (Fig. 5). This result is consistent with down-regulation of both FUMH and MDHM enzymes reported in Table 1. Clearly, the decrease in malate production ultimately reversed the malate-aspartate shuttle (Fig. 6), as well as cGMP concentration was decreased (Panel 6 A) (see discussion).

**Figure 7:**
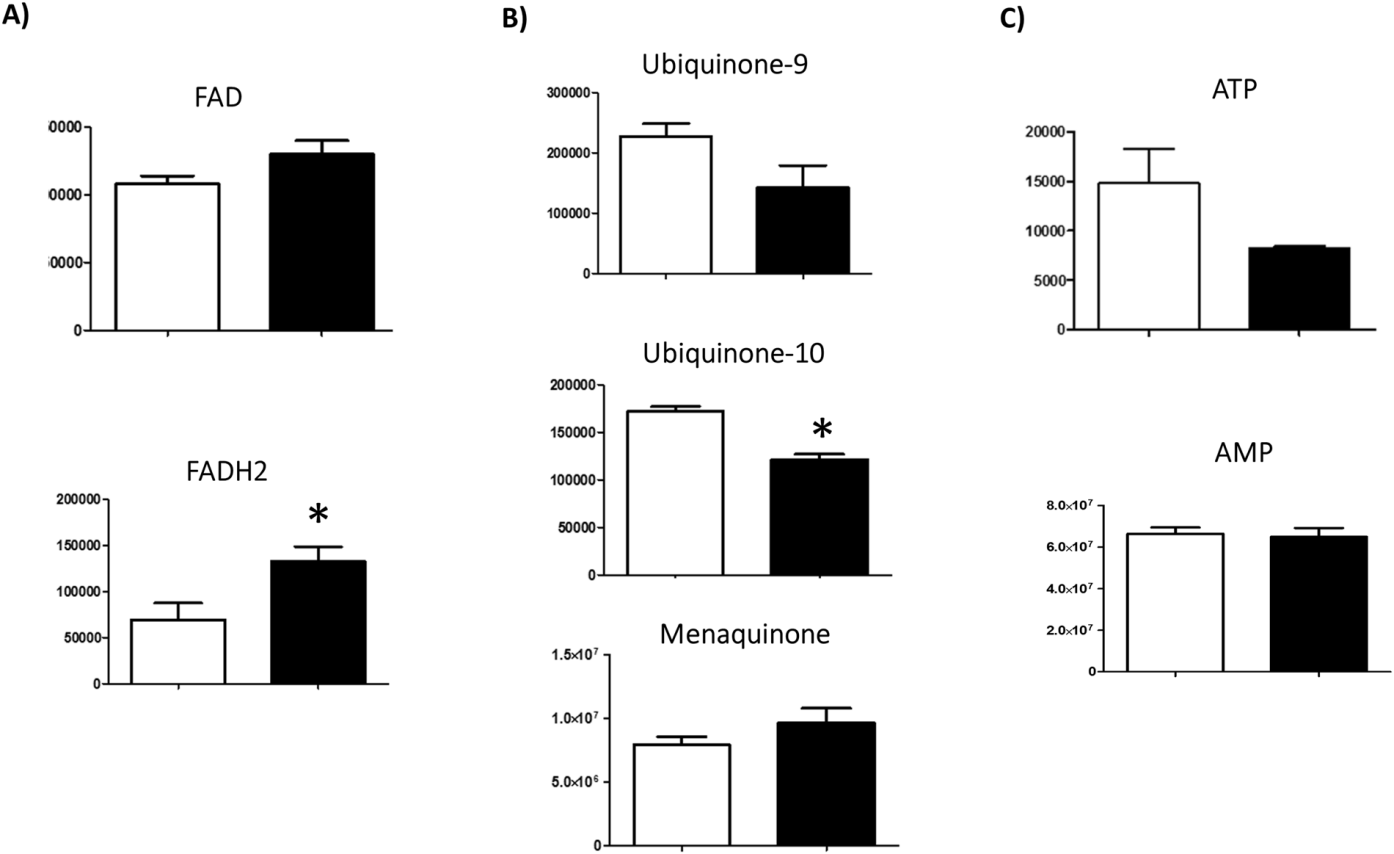
Cellular levels of FAD, Quinones, ATP. Variation in the levels of metabolic intermediates in (**A**) FAD/FADH_2_, (**B**) ubiquinone 9, ubiquinone 10 and menachinone, (**C**) ATP and AMP. Values are mean ± SD (n = 9) of normogravity (white columns) and microgravity (black columns) metabolites. Statistical significance was indicated by *p < 0.05; **p < 0.01; ***p < 0.001.

Finally, Fig. 7 shows some interesting metabolites, such as FAD and FADH_2_, ATP and AMP, quinone 9 and 10, menaquinone and IP3, whose variations with exposure to microgravity will be discussed later. Interestingly, FADH2 increased with respect to FAD (Fig. 7A), while, quinone 9 and 10 decreeased (by 40%; Fig. 7B), as did, ATP (by >45%; Fig. 7C).

With regard to amino acids, significative changes were observed for glutamic and aspartic acids which participate in the malate-aspartate shuttle, no additional significant changes were recorded, with the exception of serine (Supplementary Fig. S5).

## Discussion

Proteomic and metabolomic analyses performed on human primary osteoblasts exposed to simulated microgravity for five days enabled us to improve understanding of the responses of human osteoblasts to microgravity and to shed light on the risks associated with extended travel beyond Earth’s orbit.

The dominant effect of microgravity was disruption of osteoblast mitochondrial function, as previously observed in skeletal muscle tissue^21^, cardiomyocytes^23^ and plants^25^. These similar effects, although occurring in different species, suggest that mitochondrion changes could be an adaptative response to ensure sufficient cell energy for facing microgravity^23^.

Metabolomic analysis showed an “interruption” of the Krebs cycle at the fumarate production step, related to decreased expression of succinate dehydrogenase (Complex II of the mitochondrial respiratory chain), as confirmed by proteomic analysis (Table 1). This enzyme complex is involved in both the Kreb’s cycle and the mitochondrial respiratory chain, catalysing succinate-fumarate transformation in the Kreb’s cycle generating FADH_2_ from the prosthetic group FAD, which feeds electrons to Complex II. Interestingly, with microgravity exposure, osteoblasts showed a significant increase in menaquinone (Fig. 7B), which is an essential component of FAD^26^ even though human electron transport chain does not metabolise it. Since, it is recognised that osteoporosis in astronauts can be prevented by menaquinone supplement^27,28^, we can speculate that a correlation might exist between effects of microgravity on the respiratory chain and effects on succinate dehydrogenase.

**Figure 8:**
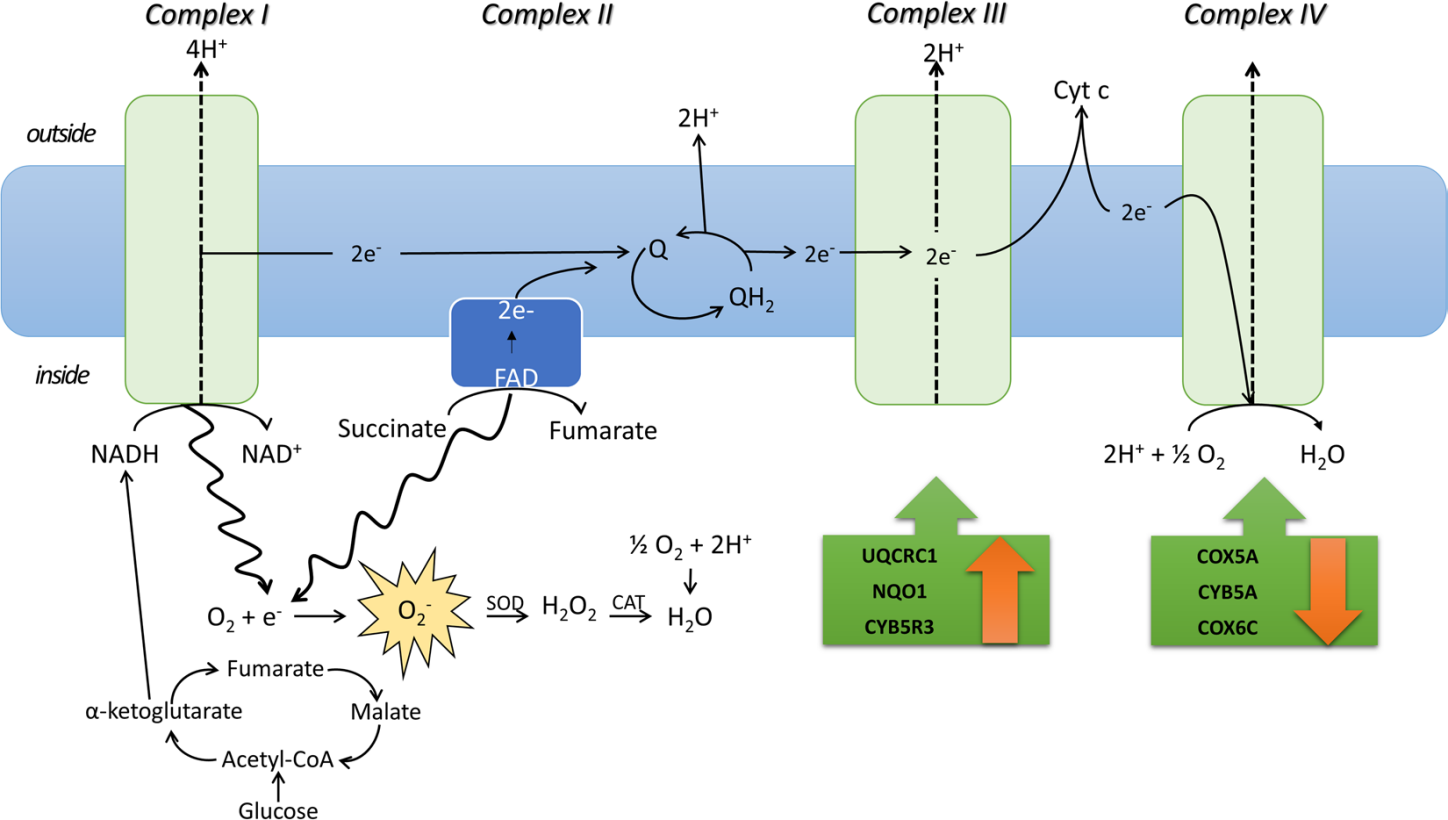
Crosstalk between TCA cycle and Mitochondrial electron transport chain in microgravity. Succinate accumulation shows toxic effect of reactive oxygen species from mitochondrial electron transport chain. An unbalanced proton pump could be associated with FADH_2_ accumulation. Green boxes show enzymes’ trend (statistical significance is shown in Table 1).

It is interesting to underline that the accumulation of succinate is one of the metabolic mitochondrial responses to extreme environments, supporting a concerted down-regulation of Kreb’s cycle and electron transport chain activity^29^. For example, in the cellular response to ischemia, succinate accumulation showed a toxic effect, which has been directly linked to mitochondrial reactive oxygen species production from Complex I (Fig. 8)^30^. Thus, it is not surprising that mitochondrial Complex II deficiency is one of the rarest disorders of the oxidative phosphorylation system (OXPHOS) which could lead to clinical disturbances, accounting for 2–8% of cases of mitochondrial disease^31,32^.

Proteomic analysis also showed dysfunction of the mitochondrial membrane caused by impairment of the other mitochondrion respiratory chain components. Total Complex III were over-expressed (by more than 60%), related to the significant over-expression of its protein components, reported in Fig. 8 and Table 1. Moreover, since the complete cycle between Complex II and III involves two electrons being transferred from ubiquinol to ubiquinone via two cytochrome c intermediates, it is unsurprising that microgravity resulted in decrease in the amount of coenzymes Q9 and Q10, as well as in the proton gradient (Fig. 7B). Primary coenzyme Q10 deficiency has been included among the mitochondrial respiratory chain disorders because of its central role as an electron carrier from Complex I and II to Complex III^33^. More than 75% of the body’s energy is a result of the role of coenzyme Q10 in mitochondrial production of ATP. Thus, people with low levels of this coenzyme may feel tired upon walking, or exhausted after just a few minutes of walking^34^. It is tempting to speculate that the recorded decrease of ATP levels (Fig. 7C), probably linked to the down-regulation of CoQ10^35^, and justifies the fatigue observed in astronauts^36^.

Recently, an increase in Complex III in human embryonic kidney cells was reported to be related to increased mitochondrial membrane potentials and enhanced cell survival under conditions of oxidative stress^37^. This agrees with our proteomic and metabolomic analyses. In fact, during reactions between Complex III and IV, a small fraction of electrons leaves the electron transport chain, thus in the presence of oxygen the premature electron leakage generates superoxide species (O_2_−). Since Complex III produces the membrane-permeable superoxide HOO^.^ rather than the membrane-impermeable O_2−_^38^, this can be released into both the mitochondrial matrix and the intermembrane space, and thus can easily reach the cytosol^39^. ROS are highly toxic and are thought to play a role in several pathologies, as well as in ageing^40^, and mitochondrial ROS have been reported to increase under microgravity conditions, bringing about mitochondrial dysfunction in rat cerebral arteries^41^. Interestingly, metabolomics analysis reported an increased amount of GSSG, while with microgravity proteomics showed up-regulation of antioxidant enzymes, such as SODC, GPX7, GPX8, and PRDX 4, 5, and 6 (Table 1).

Finally, cytocrome c oxidase (CytOx) activity (Complex IV) was reduced by 14%, as a consequence of a reduction in proteins belonging to Complex IV that were found to be significantly under-expressed (see Fig. 8 and Table 1). As a final result, ATP synthesis coupled to proton transport and cytoskeleton proteins were down-regulated as well as ATP concentration (Fig. 7C). Since the synthesis of ATP in healthy mitochondria is facilitated by the malate-aspartate shuttle system in order to restore the concentration of NADH in matrix, it is not surprising that under microgravity this shuttle system works in reverse (Fig. 6).

It is interesting to underline that reduction in CytOx by microgravity is tissue-specific, as previously reported for Complex II. Differences exist in the regulation of nuclear-encoded mitochondrial proteins in heart compared to skeletal muscle: in skeletal muscle CytOx was significantly reduced by 41%, while in cardiac muscle it remained unchanged^42^.

Finally, MetaboloAnalyst results showed interesting information: D-galactose (D-gal) metabolism is strongly altered by microgravity (Supplementary Fig. S4). Recently, in rodents it was demonstrated that chronic administration of D-gal increased the activity of the respiratory chain Complex I in the prefrontal cortex and hippocampus. Also, the activity of Complex II-III increased with oral D-gal treatment^43^. In agreement with these findings, oral administration of D-gal appeared to induce alterations in the mitochondrial respiratory complexes observed in brain neurodegeneration^44^. Thus, with exposure to microgravity a relationship between D-galactose metabolism and respiratory chain proteins occurs.

Consequently, to perturbation of mitochondrial chain reaction enzymes, induced adaptations of mitochondrial metabolisms. Glycolysis was stimulated in agreement with previous investigations, namely that during spaceflights microgravity increased glycolytic enzymes in rats^45^. This agrees with the significant increase of lactate (69%) reported here and its role in mitochondrial dysfunction.

The impairment of succinate-fumarate reaction, catalysed by Complex II, as well as the down-regulation of MDHM enzyme (Table 1), reduced malate production and, as a result, the aspartate-malate shuttle was reversed (Fig. 6). This contributed to, or is a consequence of, reduced ATP synthesis, since, after ATP synthesis, the concentration of NADH in the mitochondria (which cannot cross the mitochondrial membrane) needs to be restored in the matrix by the malate-aspartate shuttle. Thus, there is a strict correlation between ATP synthesis, MDHM activity, NADH and cGMP production. This was confirmed by treatment of ischemic cardiomyocytes^46^ with sildenafil: MDHM activity increases as well as the cGMP concentration and the malate-aspartate shuttle activity. Clearly, with microgravity this phenomenon is magnified by a reduced electron transport chain.

With respect to lipid metabolism, microgravity exposure also increased lipid synthesis in the skeletal system, as observed in the fatigued muscle cells^34^, which shifts from lipid toward glucose metabolism. The reduced glycerol shuttle, increased triglycerides production, shifting metabolism toward a higher reliance on lipids^47^. The increased lipid production was also paralleled by a reduction in beta oxidation of fatty acids inside the mitochondria, as indicated by a significant increase in acyl-CoA concentration and the concomitant decrease of acyl-carnitine (Fig. 3), both of which are needed to activate or translocate fats into the mitochondria. This is in agreement with these results, Baldwin *et al*.^48^, where an increased reliance on glycolysis coupled with a reduced ability to oxidise fats contribute to fatigue of muscle cells^49–51^, with accumulation of lactic acid. Moreover, accumulation of lipids increases demineralization of bone, which, in addition to the increased oxidative stress (documented above), inhibits differentiation of osteoblasts and stops the mineralization process of bone.

Overall, increased glycolysis and alterations in respiratory chain reactions, as well as changes in some metabolic pathways, are probably responsible for the subsequent microgravity-dependent effects (well documented in literature), such as pro-apoptotic state and cell dedifferentiation^11,12^, which will be discussed in a next manuscript. In this regard, it was recently found that alteration of mitochondrial cytochrome bc1, a component of the electron transport chain Complex III, leads to activation of tumor suppressor p53, followed by apoptosis induction^52^. Recently, it has also been observed that up-regulation of glycolytic genes and down-regulation of mitochondrial genes, as well as a marked increase in succinate, trigger the induction of IL-1β via HIF-1in macrophages^53^.

Clearly, all these aspects taken together are complex and merit further study which will be the subject of a subsequent report. However, taken together, our results indicate impairment in the physiological functions of mitochondria as well as impairment in osteoblast functionality as a specific effect of stress caused by exposure to microgravity.

## Methods

### Simulated microgravity

The desktop Random Positioning Machine (RPM) system (Dutchspace, The Netherlands) was used for conducting examinations on the influence of the force of gravity on eukaryotic cells^54^. All experiments were carefully planned according procedures previously described^55^. Briefly, the rotating frame of the desktop RPM was placed inside an ordinary cell culture CO_2_ incubator. The software responsible for controlling the motion of RPM employed a tailored algorithm, which rotated with a random speed in such a way that the mean gravity vector reliably converged to zero over time, and it concurrently reduced fluid motion in the culture flask. In order to avoid artifacts and to minimise centrifugal acceleration, the samples were compactly placed around the center of rotation. Cell samples were carefully processed for *in vitro* cultivation: the culturing media were accurately sealed with a transpiring membrane, which was pressed to completely remove air bubbles from the culture chamber. Control samples were processed in the same manner. Plates were placed beside the RPM machine so that all samples shared identical culture conditions.

### Patient characteristics

Patients selected for the study were screened to exclude any associations with clinical or pathological variables (carefully grouped on the bases of BMD parameters obtained by dual-energy X-ray absorptiometry (DEXA) T-score greater that -1). No patients showed any signs of bone or joint disease or autoimmune disorder. Informed consent was obtained from all patients. The biopsies were collected from high energy fractures of femoral head of healthy patients during hip replacement surgery. The bioptic samples from selected patients were taken to set up primary cells and human primary cells were cultured *in vitro*. All procedures were approved by the Institutional Review Board of Policlinico Tor Vergata Hospital, Rome, Italy (approval reference number # 85/12). Since no effects of sex on adaptation to space had been previously observed^56^, we decided to analyse osteoblasts from both male and female patients in order to determine average effects.

Experiments were performed in accordance with relevant guidelines and regulations. Human primary osteoblasts (hOBs) were isolated from 3 donors (2 male and 1 female; average age 53 years; female 52 years, males 49 and 59 years) undergoing hip arthroplasty surgery and used to perform separate experiments investigating the effects of microgravity *versus* normogravity conditions.

### Isolation and Culture of Primary Human OB Cells

Primary cultures of osteoblasts were isolated from the cancellous bone of healthy patients with high-energy femoral fracture. The bone tissue was minced, thoroughly washed to remove any remaining soft tissue, and placed in 6-well plates to initiate explant cultures. The culture medium consisted of DMEM/F12 (DMEM w/o L- glutamine w/ 25 mM Hepes, Biowest, Nuaillé, FR.) supplemented with 15% FBS, 50 μg/mL gentamicin and 0.08% Fungizone, penicillin streptomycin (Sigma Chemical Co., St Louis, MO, USA), and amphotericin B (biowest) and was changed twice per week. Cells were treated to select and isolate homogeneous populations of osteoblasts according to previously reported methods^57^. The Supplementary Information provide details of instrument settings.

### Osteoblast characterisation

Primary osteoblasts were cultured for few weeks in osteogenic medium (OGM) containing 10 mM biglycerol phosphate, 50 µM ascorbic acid, 25 ng/ml bone morphogenetic protein-2 (BMP-2; R&D Systems) in 10% serum containing alpha MEM. After two weeks of incubation, the cells were assessed for alkaline phosphatase activity as an indicator of differentiation. Morphological inspection was carried out to assess HA precipitated crystals. Osteoblast phenotype was characterised by immunohistochemistry as BMP-2, RUNX-2 and RANK-L (paper in preparation).

### Assays to evaluate the effect of simulated microgravity on cell number

Cells were seeded in a monolayer at 18 000 to 40 000 cells/cm^2^ and cultured until the confluence was reached. When required, cells were detached by incubating plates with trypsin/versine solution for 10 minutes at 37 °C. The number of viable cells were assessed using the trypan blue dye exclusion procedure. Cell solution was mixed 1:1 with trypan blue stain (Invitrogen) and cells with intact membranes (viable cells) were counted using a haemocytometer device under an optical microscope (Nikon Eclipse TE 2000–5). Counts were performed in twice and repeated for three biological replicates. To determine the number of viable metabolically active cells (i.e. possessing active mitochondria), the cell titer 96 AQ cell colorimetric assay (Promega) was employed, following the manufacturers’ instructions. After incubation for four hours, MTS formazan soluble product was measured at absorbance 490 nm with a Tecan Spark 10 N spectrophotometer reader. Spectrophotometric determination of cell protein content was evaluated by the biuret colorimetric analysis of peptides and the bicinchoninic acid (BCA) method, with readings at wavelengths of 310 nm and 562 nm, respectively (Biuret reagent kit, Sigma-Aldrich; BCA Protein Assay Kit, The Thermo Scientific Pierce).

For all three assays, standard curve validated that the absorbance (at 490 nm, 310 nm or 562 nm, respectively) read were directly proportional to the number of living cells in culture. The effect of the treatment on the number of cells was reported in term of percentage, as follows: the percentage calculation was based on the ratio of the mean value of microgravity-treated samples to the mean value of normo-gravity (control) samples. The statistical significance of data was determined using the Student’s t-test.

### MS sample preparation

On day 5, microgravity-treated and control cells (3 biological replicates) were washed in phosphate saline buffer. Cellular suspensions were centrifuged at 1500 *g* for 5 min. The supernatant was discarded and the cell pellet was resuspended in lysis buffer (7 M urea, 2 M tiourea, 4% w/v CHAPS, 40 mM Tris-HCl, 0.1 Mm EDTA, 1 mM DTT, 50 mM NaF, and 0.25 mM Na_2_VO_4_).

The 2D-Quant Kit (GE Healthcare) was used for determination of total protein concentration. Aliquots (150 µg) of each sample were loaded per lane and separated through a 16–8% linear gradient polyacrylamide gel. Each lane was cut into 72 slices, approximately 2-mm thickness; these were subjected to in-gel trypsin-digestion according to the protocol of Schevchenko *et al*.^58^.

### LC-MS/MS analysis

Peptide extracts were analysed using a split-free, nano-flow liquid chromatography system (EASY-nLC II, Proxeon, Odense, Denmark) coupled with a 3D-ion trap (model AmaZon ETD, Bruker Daltonik, Germany), equipped with an online ESI nanosprayer (fused-silica capillary, 0.090 mm OD, 0.020 mm ID) in positive ion mode. Details of instrument settings are provided in Supplementary Information.

### MS data analysis

Compass DataAnalysis 4.0 software (Bruker Daltonics) was used for data processing. Generated MGF files were then merged per lane and used in a database search (SwissProt, version 20150612), using the Mascot Daemon application included in an in-house MASCOT server (version 2.5, Matrix Science, London, UK) with the following constraints: taxonomy = Homo sapiens (20207 sequences); enzyme = trypsin; missed cleavage = 1; peptide and fragment mass tolerance =  ± 0.3 Da; fixed modifications = carbamidomethyl (Cys); variable modifications = oxidation (Met).

Label-free quantitative analyses were performed on three biological triplicates using the spectral counting method based on normalised exponentially modified protein abundance index (emPAI), as described by Shinoda *et al*.^59^.

To obtain a comprehensive description of the over-represented biological processes and functionally-related groups of proteins within our dataset, a Bioinformatic Gene Ontology analysis was performed using the on-line FunRich (Functional Enrichment analysis tool) software 3.0 (www.funrich.org). The default Homo sapiens genome was used as background.

### Metabolomic extraction

For each treatment, 1 * 10^6^ cells (3 biological replicates × 3 technical replicates × 2 conditions; n = 18) were first subjected to three freeze-melt cycles (freezing in ice at 4 °C for 5 min, melting at 37 °C for 5 min; for 5 times). Next, 400 μl of freezing methanol and 600 μl of freezing chloroform were added to the cells. Samples were vortexed for 30 min at max speed at 4 °C. The next day, samples were centrifuged at 16000 *g* for 15 min at 4 °C. Supernatants were then evaporated to dryness using an SPD2010–230 SpeedVac Concentrator (Thermo Savant, Holbrook, USA). When samples were completely dried, 60 μl of 5% formic acid was added to the dried residue and vigorously vortex-mixed.

### UHPLC-HRMS

Twenty microliters of each sample was injected into an Ultra High-Performance Liquid Cromatography (UHPLC) system (Ultimate 3000, Thermo) and run on a Positive mode. Samples were loaded onto a Reprosil C18 column (2.0 mm × 150 mm, 2.5 μm; Dr Maisch, Germany) for metabolite separation. The Supplementary Information provides details of instrument settings.

## Data elaboration and statistical analysis

Replicates were exported as mzXML files and processed using MAVEN 5.2. Mass spectrometry chromatograms were elaborated for peak alignment, matching and comparison of parent and fragment ions, and tentative metabolite identification (within a 2ppm mass-deviation range between observed and expected results against the imported KEGG database). Untargeted metabolomic profiling of cells from two independent cohorts of treated and control cells was conducted to reduce noise in the data analysis. Pre-processing was performed to exclude xenobiotics, carbohydrates, and metabolites with more than a 3-fold difference to the control group (*p* < 0.01).

Metabolites detected across both groups that met acceptability criteria and were further analysed using MetaboAnalyst 3.0 software. Metabolite set enrichment analysis (MSEA) and pathway analysis (MetPA) were used to determine the biological processes involved in the conditions of interest. Both have been developed to identify and to interpret patterns in concentration changes of human osteoblast metabolites under conditions of microgravity.

The metabolic pathways affected the most by the “weightlessness” treatment in human osteoblast cells were screened by MetPa, assessing the differential impact of metabolites on the overall metabolism (the pathway library was restricted to Homo sapiens and *p*-value < 0.05).

The difference between the two groups was compared using the unpaired t-test with GraphPad Prism version 5.0 GraphPad software (La Jolla Ca); *p < 0.05 was considered significant.

## Electronic supplementary material

supplemetary information
