# Supplementary material for: Effects of microgravity on osteoblast mitochondria: a proteomic and metabolomics profile
Source: Sci Rep. 2017 Nov 13;7:15376. doi: 10.1038/s41598-017-15612-1 (PMC5684136; doi:10.1038/s41598-017-15612-1)
Supplement: Supplementary file 1 — supplemetary information [file 41598_2017_15612_MOESM1_ESM.pdf]

Effects of microgravity on osteoblast mitochondria: a proteomic and metabolomics profile

Anna Michaletti[1], Magda Gioia[2], Umberto Tarantino[2] and Lello Zolla[1] *

1 Department of Ecological and Biological Sciences, University of Tuscia, Viterbo, Italy.

2 Department of Clinical Medicine and Translational Science, University of Rome Tor Vergata, Rome Italy.

* Corrisponding author:

Lello Zolla: zolla@unitus.it

Supplementary methods

Isolation and Culture of Primary Human OB Cells

Primary cultures of osteoblasts were isolated from the cancellous bone of health patients with high-energy femoral fracture. The bone tissue was minced, thoroughly washed to remove any remaining soft tissue, and placed in 6-well plates to initiate explant cultures. The culture medium consisted of DMEM/F12 (DMEM w/o L- glutammine w/ 25mM Hepes, Biowest, Nuaillé, FR.) supplemented with 15% FBS, 50 µg/mL gentamicin and 0.08% FungizoneR, penicillin streptomycin (sigma Chemical Co.,St Louis, MO, USA), amphotericin B (biowest) and was changed twice per week). Cells were treated to select and isolate homogeneous population of osteoblasts according previously established methods[1]. Briefly, after dissection, trabecular bone chips were repeatedly washed with PBS at 37°C for 2 h in shaking conditions. Then, two distinct enzymatic digestions were repeated and performed at 37°C. The first digestion employed 1mg/ml Trypsin from porcine pancreas ≥ 60 U/mg (SERVA Electrophoresis GmbH Heidelberg, DE) resuspended in PBS buffered at pH 7.2. After washing, trypsinized bone chips underwent to repeated digestions with a second type of protease employing 2.5 mg/ml Collagenase NB 4G Proved grade ≥ 0.18U/mg (SERVA Electrophoresis GmbH, Heidelberg, DE) in PBS buffer with Calcium and Magnesium. The supernatants from the 4th bone-chips digestion was collected and centrifuged at 310 RCF for 5'. The cell pellets were suspended in DMEM with 15% FBS, thus cells were then grown in low calcium media, supplemented with fetal bovine serum (10%; Intergen, Purchase, NY, USA), penicillin (50 U/ml), and streptomycin *(50* pg/ml). When the cultures reached confluence in 3-5 weeks, the bone chips were removed and the cellular outgrowths treated with trypsin (0.05%) and EDTA (0.02%) to prepare single cell suspensions. All cells were incubated at 37°C and 5% CO2. Upon confluence, cells were detached from the plates by trypsinization, counted and subcultured at a density of 5000 cells/cm2 for three passages. Osteoblast proliferation was compared between different tissue sources at passage one. Third passage cells were used in all other experiments.

LC-MS/MS analysis.

Peptide extracts were analyzed using a split-free nano-flow liquid chromatography system (EASY-nLC II, Proxeon, Odense, Denmark) coupled with a 3D-ion trap (model AmaZon ETD, Bruker Daltonik, Germany) equipped with an online ESI nanosprayer (the spray capillary was a fused silica capillary, 0.090 mm OD, 0.020 mm ID) in positive ion mode. For all experiments, a 15 µL sample volume was loaded by the autosampler onto a homemade 2 cm fused silica precolumn (100 µm I.D.; 375 µm O.D.; Reprosil C18-AQ, 5 µm, Dr. Maisch GmbH, Ammerbuch-Entringen, Germany). Sequential elution of peptides was accomplished using a flow rate of 300 nL/min and a linear gradient from Solution A (2% acetonitrile; 0.1% formic acid) to 50% of Solution B (98% acetonitrile; 0.1% formic acid) in 40 min over the precolumn on-line with a homemade 15 cm resolving column (75 µm I.D.; 375 µm O.D.; Reprosil C18-AQ, 3 µm, Dr. Maisch GmbH, Ammerbuch-Entringen, Germany). The acquisition parameters for the mass spectrometer were as follows: dry gas temperature, 220 °C; dry gas, 4.0 L/min; nebulizer gas, 10 psi; electrospray voltage, 4000 V; high-voltage end-plate offset, −200 V; capillary exit, 140 V; trap drive: 63.2; funnel 1 in 100 V out of 35 V and funnel 2 in 12 V out of 10 V; ICC target, 200,000 and maximum accumulation time, 50 ms. The sample was measured with the Enhanced Resolution Mode at 8100 m/z per second (which allows monoisotopic resolution up to four charge stages), scan range from m/z 300 to 1500, 5 spectra averaged, and rolling average of 1. The "Smart Decomposition" was set to "auto".
Label-free quantitative analyses were performed in biological triplicates by using the spectral counting method based on normalized exponentially modified protein abundance index (emPAI) as described by Shinoda et al[2]. In detail, for each protein the following percentage was calculated:

Protein content (%) = emPAI/ΣemPAI × 100.

Statistically significant differences were identified by unpaired t-student test.

UHPLC-HRMS

Twenty microliters of samples were injected into an Ultra High-Performance Liquid Cromatography (UHPLC) system (Ultimate 3000, Thermo) and run on a Positive mode: Samples were loaded onto a Reprosil C18 column (2.0 mm × 150 mm, 2.5 µm — Dr Maisch, Germany) for metabolite separation. Chromatographic separations were achieved at a column temperature of 30 °C; and flow rate of 0.2 mL/min. For positive ion mode (+) MS analyses, a 0–100% linear gradient of solvent A (ddH2O, 0.1% formic acid) to B (acetonitrile, 0.1% formic acid) was employed over 20 min, returning to 100% A in 2 min and a 6-min post-time solvent A hold. Acetonitrile, formic acid, and HPLC-grade water and standards (≥98% chemical purity) were purchased from Sigma Aldrich. The UHPLC system was coupled online with a mass spectrometer Q Exactive (Thermo) scanning in full MS mode (2 µscans) at 70,000 resolution in the 67 to 1000 m/z range, target of

1´106 ions and a maximum ion injection time (IT) of 35 ms 3.8 kV spray voltage, 40 sheath gas, and 25 auxiliary gas, operated in positive ion mode. Source ionization parameters were: spray voltage, 3.8 kV; capillary temperature, 300 ° C; and S-Lens level, 45. Calibration was performed before each analysis against positive or negative ion mode calibration mixes (Piercenet, Thermo Fisher, Rockford, IL) to ensure sub ppm error of the intact mass. Metabolite assignments were performed using computer software (Maven,18 Princeton, NJ), upon conversion of raw files into.*mzXML* format through MassMatrix (Cleveland, OH).

REFERENCE

1. Siggelkow, H. *et al.* Development of the osteoblast phenotype in primary human osteoblasts in culture: comparison with rat calvarial cells in osteoblast differentiation. *J Cell Biochem*. **75**(1), 22-35 (1999).
2. Shinoda, K., Tomita,M. & Ishihama, Y. emPAI Calc–for the estimation of protein abundance from large-scale identification data by liquid chromatography-tandem mass spectrometry. *Bioinformatics*. **26**, 576–577 2010).

Supplementary Figures

A

A

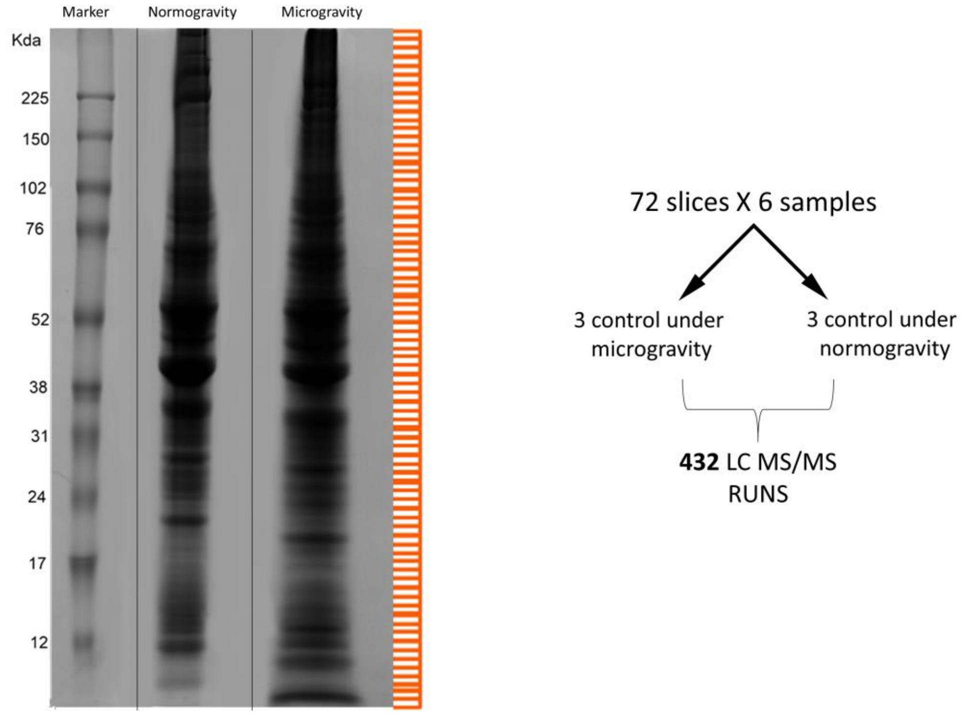

B

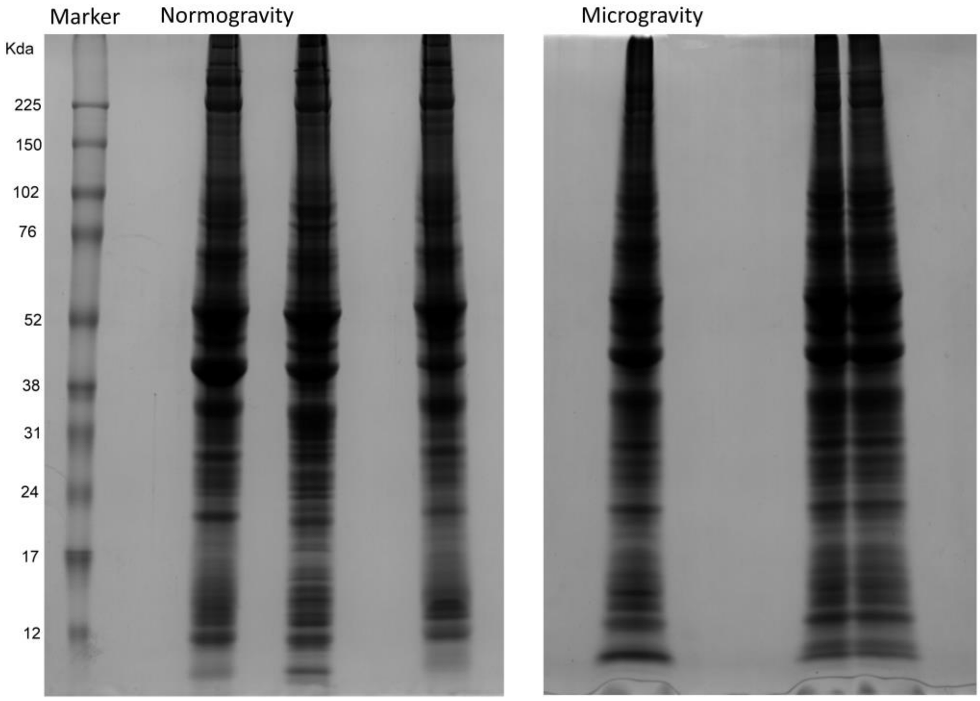

Fig. S1 One dimensional SDS-PAGE (16–8% linear gradient polyacrylamide gel). A) A 150 µg aliquot of human osteoblast proteome was loaded per lane. Three biological replicates per condition were analyzed. The gels were stained with blue-Comassie. The vertical line indicates where gel- images was cropped; the two original images are reported on panel B.

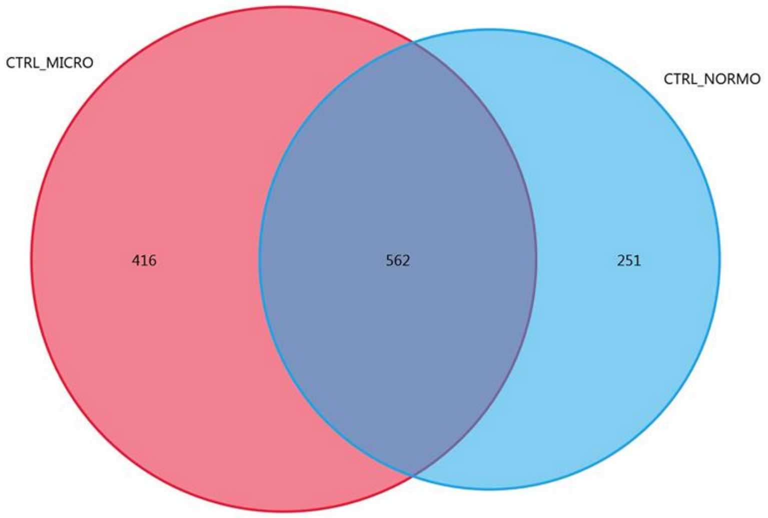

Fig. S2. **Venn Diagram.** Depicting the comparative protein profiling of human osteoblast under normogravity and under microgravity it was revelated that 251 proteins were uniquely detected in normogravity, 416 were detected exclusively in microgravity and 562 were shared.

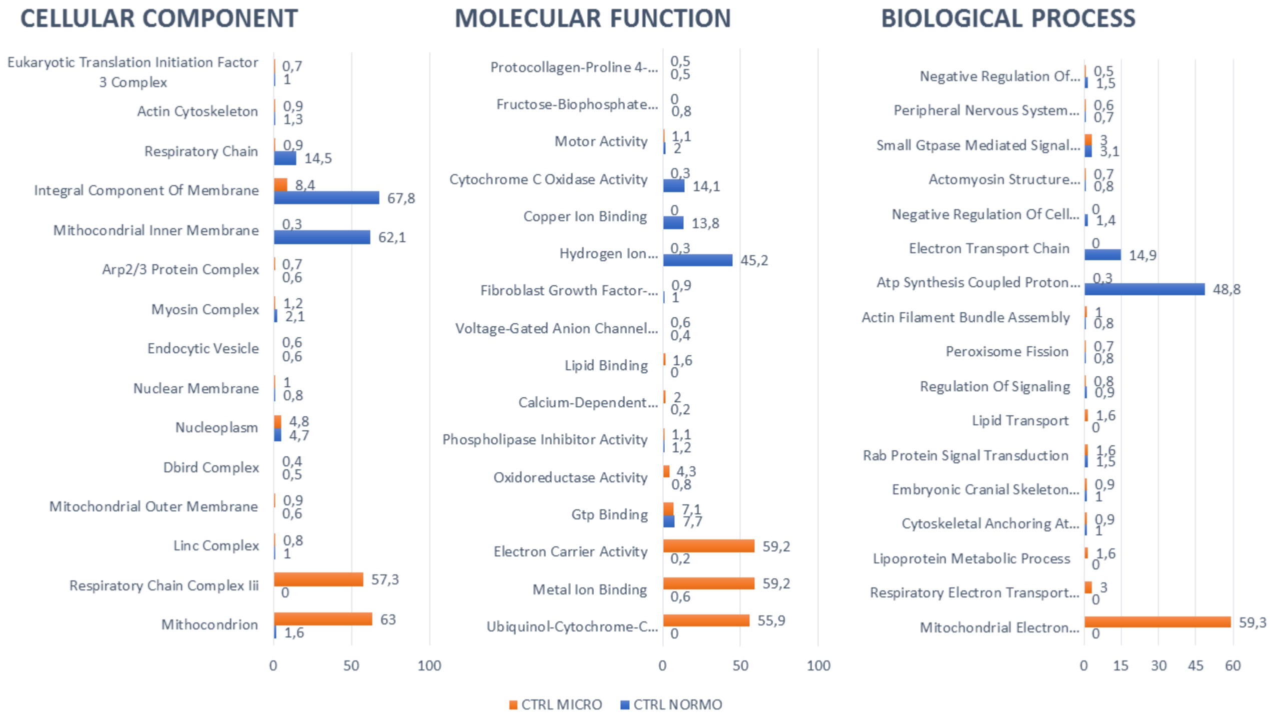

Fig. S3. Functional enrichment analysis of hpOB under Normogravity and Microgravity using FunRich. Bioinformatics Gene Ontology-based classification of proteomes according to three categories: cellular components (A), molecular function (B), and biological processes (C).

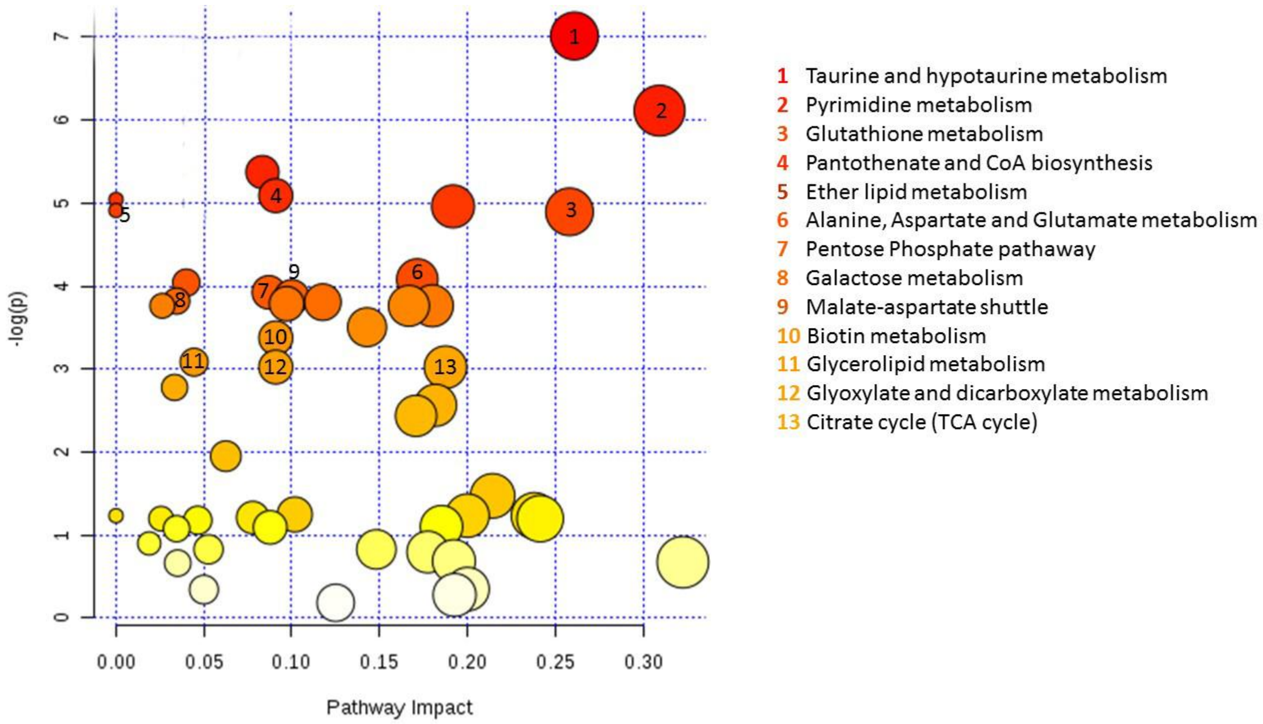

Fig. S4. **Metabolic Set Enrichment Analysis. We analyzed the MetPA plot using Metaboanalyst software**. Color intensity (light blue means the metabolite are is not in your data but used as background for enrichment analysis; grey means the metabolite is not in your data and also excluded from enrichment; other colors varying from yellow to red reflects increasing statistical significance, while circle diameter covaries with pathway impact. The graph was obtained plotting on the y-axis the –log of p-values from pathway enrichment analysis and on the x-axis the pathway impact values derived from pathway topology analysis.

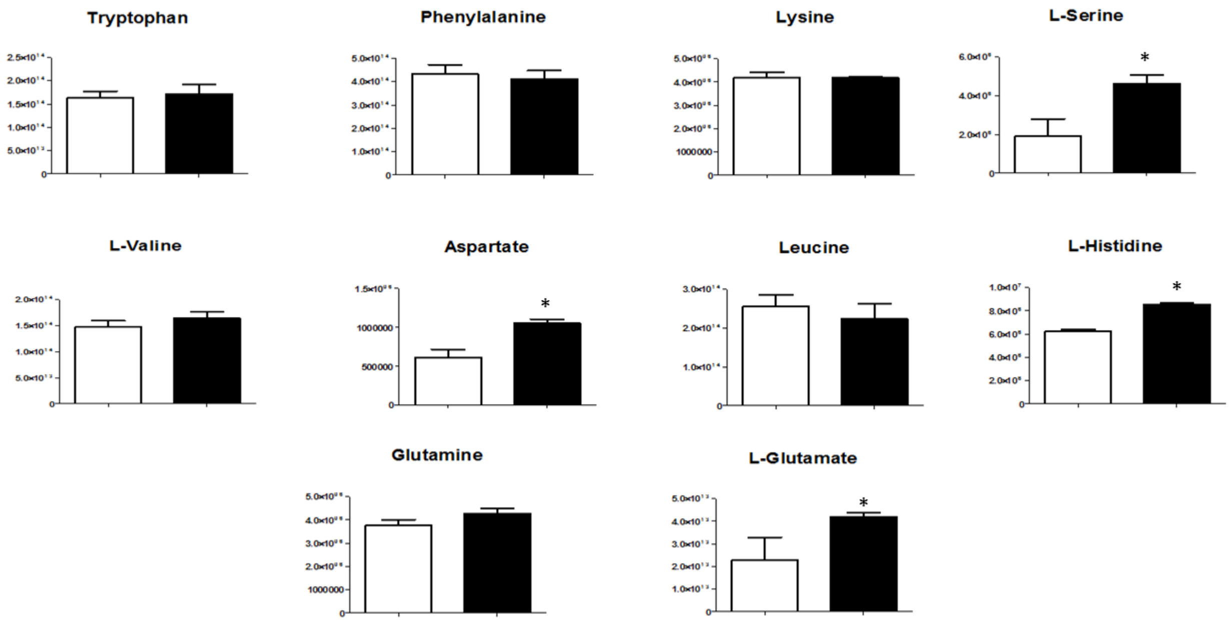

Fig. S5. **Amino acids**. The columns white represented mean ± SD (n=9) of metabolite concentration in normogravity, the column in black represented mean ± SD (n=9) of metabolite concentration in microgravity. Statistical significance was indicated with *p< 0.05; **p< 0.01; ***p< 0.001.
